# Cardiometabolic Comorbidities in Patients With Psoriasis: Focusing on Risk, Biological Therapy, and Pathogenesis

**DOI:** 10.3389/fphar.2021.774808

**Published:** 2021-11-04

**Authors:** Jiangluyi Cai, Lian Cui, Yu Wang, Ying Li, Xilin Zhang, Yuling Shi

**Affiliations:** ^1^ Department of Dermatology, Shanghai Skin Disease Hospital, Tongji University School of Medicine, Shanghai, China; ^2^ Institute of Psoriasis, Tongji University School of Medicine, Shanghai, China; ^3^ Department of Dermatology, Shanghai Tenth People’s Hospital, Tongji University School of Medicine, Shanghai, China

**Keywords:** psoriasis, biologics, cardiovascular disease, diabetes mellitus, obesity, metabolic syndrome

## Abstract

Psoriasis is a chronic inflammatory disease characterized by erythematous scaly plaques, accompanied by systemic damage that leads to the development of multiple comorbidities. In particular, the association between psoriasis and cardiometabolic comorbidities, including cardiovascular diseases (CVDs), obesity, diabetes mellitus, and metabolic syndrome, has been verified in a considerable number of clinical trials. Moreover, the increased risk of cardiometabolic comorbidities positively correlates with psoriasis severity. Biologic therapy targeting inflammatory pathways or cytokines substantially improves the life quality of psoriasis patients and may affect cardiometabolic comorbidities by reducing their incidences. In this review, we focus on exploring the association between cardiometabolic comorbidities and psoriasis, and emphasize the benefits and precautions of biologic therapy in the management of psoriasis with cardiometabolic comorbidities. The pathogenic mechanisms of cardiometabolic comorbidities in psoriasis patients involve common genetic factors, lipid metabolism, insulin resistance, and shared inflammatory pathways such as tumor necrosis factor-*α* and interleukin-23/Th-17 pathways.

## Introduction

Psoriasis is a chronic immune-mediated inflammatory disease characterized by erythematous scaly plaques that commonly develop on extensor surfaces. Its histopathological features are hyperkeratosis, parakeratosis, epidermal acanthosis, and the infiltration of immune cells. Psoriasis has been estimated to affect 2–3% of the world population and has deleterious effects on the quality of life of patients (Griffiths and Barker, 2007).

With recent advances in the understanding of psoriasis, the disease is increasingly being considered as a systemic inflammatory disorder rather than only involving the skin and joints. An increased risk of inflammatory comorbidities such as cardiovascular diseases (CVDs) and metabolic diseases, collectively called cardiometabolic comorbidities, has been reported in psoriasis patients. There is increasing awareness that cardiovascular risk factors would enhance the potential risks of cardiovascular morbidity and mortality in affected psoriasis patients (Neimann et al., 2006). Psoriasis is also typically related to metabolic diseases including obesity, diabetes mellitus, and metabolic syndrome, which manifests as a combination of central obesity, hypertension, insulin resistance, and dyslipidemia (Johnsson et al., 2012). The incidences of major adverse cardiovascular events (MACEs), a composite endpoint comprised of myocardial infarction, cerebrovascular accident, and cardiovascular death, have been reported to be higher in patients with severe psoriasis (Abuabara et al., 2010; Mehta et al., 2010; Ahlehoff et al., 2012). Indeed, with an increase in the severity of psoriasis, patients are prone to a higher risk of CVDs, suggesting that the prevalence of cardiovascular events positively correlates with psoriasis severity. Analogous positive trends have also been identified between metabolic disorders and psoriasis independent of obesity and other risk factors (Yeung et al., 2013).

There has been a continuous innovation in the management of psoriasis along with the advances in understanding of its pathogenic mechanisms and essential inflammatory pathways (Boehncke and Schön, 2015). Targeted immunotherapy, including the agonists of tumor necrosis factor-*α* (TNF-*α*), interleukin-(IL)-17 and IL-23, has achieved great success (Mahil et al., 2016). First-generation biologics involves a group of TNF-*α* inhibitors, including monoclonal antibodies adalimumab, infliximab, certolizumab-pegol, and golimumab as well as its fusion protein etanercept. Second-generation biologics are composed of IL-12/23 and IL-17 inhibitor family: anti–IL-12/23p40 antibody ustekinumab; anti–IL-23p19 antibodies guselkumab, risankizumab, and tildrakizumab; anti–IL-17A antibodies secukinumab and ixekizumab; and anti–IL-17 receptor a antibody brodalumab (Rønholt and Iversen, 2017). They have now become prevalent regimens for the induction and maintenance of curative effect in patients with severe psoriasis that a higher proportion of patients have achieved Psoriasis Area Severity Index 75 (PASI 75) and PASI 90 after long-time treatment (Papp et al., 2005; Reich et al., 2005; Saurat et al., 2008). Their rapid efficacy has greatly improved the life quality of psoriasis patients (Rønholt and Iversen, 2017).

Because of the frequent association between psoriasis and cardiometabolic disorders, the impact of psoriasis therapy on the risk of cardiometabolic comorbidities have aroused our attention. In the clinical trials aimed at investigating the efficacy and safety profile of biologic therapies for psoriasis, the number of MACE cases was generally higher in the biologic groups than in the placebo groups. However, the intergroup difference was not statistically significant. This prompted us to recognize the cardiovascular risk during the medication of biologics in psoriasis patients (D’Adamio et al., 2019). TNF-*α* inhibitors reduce the risk of cardiovascular events and improve the cardiovascular outcome in patients with psoriasis, (Lee et al., 2019a). IL-17A, another important biologic target of psoriasis, plays a vital role in the pathogenesis of both psoriasis, and atherosclerotic plaques that accumulating evidence supports a beneficial influence of its agonists on related cardiometabolic comorbidities (Roubille et al., 2015; Lockshin et al., 2018). On the other hand, cardiometabolic comorbidities also affect the therapeutic effects of biologics (Boyd and Kavanaugh, 2015). Therefore, the impact of biologic therapies on cardiometabolic comorbidities should not be ignored during the treatment of psoriasis.

In this review, we explored the association between biologics and cardiometabolic comorbidities in patients with psoriasis to determine optimal systemic management of psoriasis by early screening and intervention for cardiometabolic comorbidities. We discuss the overlapping mechanisms between psoriasis and associated cardiometabolic comorbidities, especially the shared inflammatory pathways between psoriasis and cardiometabolic diseases linked to a sequence of inflammatory cascade reactions driven by increased T helper 1 (Th1), Th17 lymphocytes and associated proinflammatory cytokines, including TNF-*α*, IL-1*β*, IL-17 and IL-23 (Davidovici et al., 2010). Other overlapping pathogenic mechanisms between psoriasis and comorbid cardiometabolic disorders have been proposed to be caused by common genetic factors (Lu et al., 2013), secretion of adipokines (Deng and Scherer, 2010; Robati et al., 2014), lipoprotein particles (Salahuddin et al., 2015), insulin resistance (Gyldenløve et al., 2015), angiogenesis (Malecic and Young, 2017), endothelial dysfunction (Karbach et al., 2014), and oxidative stress (Armstrong et al., 2011; Lockshin et al., 2018).

## Manuscript Formatting

### Risk of Cardiometabolic Diseases in Psoriasis

#### Psoriasis and Cardiovascular Diseases

The increased risk of MACEs in patients with psoriasis has been discussed for decades. Previous studies showed a markedly increased incidence of cardiovascular diseases among psoriasis patients (Miller et al., 2013). A prominent role of chronic inflammation in CVDs was firstly noted (Ridker et al., 1997). Subsequently, a prospective population-based cohort study in the United Kingdom discovered that there existed a higher cardiovascular risk in patients with systemic chronic inflammatory conditions including psoriasis (Gelfand et al., 2006; Ridker, 2010). The study prompted the process of defining the association between psoriasis and cardiovascular events. Gelfand et al reported that psoriasis was an independent risk factor for myocardial infarction (MI), especially in young individuals with severe psoriasis (Gelfand et al., 2006). Furthermore, a cohort study using the General Practice Research Database demonstrated that patients with severe psoriasis are at a higher risk of cardiovascular mortality after controlling for major cardiovascular risk factors, providing stronger evidence that severe psoriasis may be an independent risk factor for cardiovascular death (Mehta et al., 2010). However, the higher prevalence of conventional cardiovascular risk factors, which are comprised of smoking, diabetes, lipid abnormalities, and hypertension, in psoriasis patients has been confirmed in many observational studies (Stern, 2010). It is well-established that psoriasis increases the risk of MI and ischemic stroke, and more recent studies have linked psoriasis with an increased risk of other CVDs such as heart failure and atrial fibrillation (Ahlehoff et al., 2012; Khalid et al., 2014). The risk of cardiovascular events is also linked to a positive dose-response relationship with objectively-measured psoriasis severity and cumulative duration of psoriasis (Yeung et al., 2013; Egeberg et al., 2017).

#### Psoriasis and Obesity

For a long time, obesity has been considered as a cardiovascular risk factor along with other factors including smoking, hypertension, and hyperlipidemia. It has been shown to be more prevalent in psoriasis patients than in patients without psoriasis (Shapiro et al., 2012). The relationship between obesity and psoriasis is estimated to be interrelated that obesity correlates with an increased risk of psoriasis and psoriasis might conversely lead to the occurrence of obesity (Budu-Aggrey et al., 2019). A recent meta-analysis of prospective studies showed that the relative risk of the relevance between psoriasis risk and per 5-unit increment in body mass index (BMI), per 10-cm increment in waist circumference, per 0.1-unit increment in the waist-to-hip ratio**,** per 5 kg of weight gain was 1.19 (95% CI: 1.10–1.28), 1.24 (95% CI: 1.17–1.31), 1.37 (95% CI: 1.23–1.53), and 1.11 (95% CI: 1.07–1.16), respectively, concluding that risk of psoriasis increases with the degree of obesity measured by the four abovementioned aspects (Aune et al., 2018). As the severity of psoriasis has a positive correlation with weight gain, several studies investigated the impact of weight loss on psoriasis severity through low-energy diet or bariatric surgery (Jensen et al., 2013; Shelling and Kirsner, 2013; Debbaneh et al., 2014). They found that weight loss in overweight patients with psoriasis showed a declined trend of psoriasis severity manifested as a reduction of PASI and improved quality of life (Jensen et al., 2013; Shelling and Kirsner, 2013; Debbaneh et al., 2014). It is possible that psoriasis might develop first and bring about obesity. This view is supported by the substantial lipid abnormalities in psoriasis patients and the increased incidence of adiposity, especially central adiposity, following the development of psoriasis in children (Mallbris et al., 2006; Paller et al., 2013).

#### Psoriasis and Diabetes Mellitus

Diabetes mellitus, considered as one of the traditional cardiovascular-related risk factors that can contribute to cardiovascular morbidity and mortality, has been linked to psoriasis in many clinical trials and meta-analyses (Miller et al., 2013). In a Danish nationwide cohort study, the incidence rate of new-onset diabetes mellitus increased among patients with psoriasis compared with the population without psoriasis and was positively correlated with psoriasis severity after correcting for confounding factors such as age, sex, concomitant medication, comorbidity, and socioeconomic status (Khalid et al., 2013). This phenomenon was also identified in a meta-analysis of 44 observational studies that showed a higher risk of type 2 diabetes mellitus (T2DM) [odds ratio (OR): 2.10, 95% CI: 1.73–2.55] in patients with severe psoriasis (Wu et al., 2015). In addition to diabetes mellitus itself, its related systemic complications also show a positive relationship with moderate-to-severe psoriasis, the severity of which was objectively measured by the body surface area (BSA) affected, independently of other risk factors including obesity and smoking (Yeung et al., 2013). The risk of psoriasis development is higher in diabetes patients, which is associated with drug exposure in those treated with anti-diabetic therapies. An increasing risk of psoriasis is linked to the frequent use of insulin (adjusted OR: 1.29, 95% CI: 1.18–1.42, *p* < 0.001). On the other hand, a reduction in the risk of psoriasis is related to the frequent use of thiazolidinedione (TZD) (adjusted OR: 0.89, 95% CI: 0.81–0.98) compared with low-frequency TZD users (Wu et al., 2015). Conversely, concomitant medication during the treatment of psoriasis could also modulate the risk of T2DM incidence (Lee et al., 2014).

A Danish population-based twin study revealed that genetic and environmental factors play a role in the comorbidity of psoriasis and T2DM (Lønnberg et al., 2016). Pleiotropic susceptibility loci CDKAL1 contributes to the occurrence of psoriasis as well as diabetes mellitus (Wolf et al., 2008), which may upregulate inflammatory cytokines in psoriasis and thus promote insulin resistance, an independent risk factor of T2DM (Gelfand, 2016). A study indicated that insulin resistance or impaired insulin sensitivity exists in psoriasis patients with normal glucose tolerance, which may further result in the development of diabetes mellitus (Gyldenløve et al., 2015). Systemic inflammation may be a potential shared pathophysiologic pathway between psoriasis and diabetes mellitus. Inflammatory mediators such as TNF-*α*, IL-6, leptin, and adiponectin affect the regulation of insulin sensitivity by various interactions *via* signaling pathways between insulin receptors and cytokines or adipocytokines (Davidovici et al., 2010; Donath, 2014).

#### Psoriasis and Metabolic Syndrome

Metabolic syndrome is a comprehensive term for a cluster of interrelated metabolic disorders such as abdominal obesity, hypertension, insulin resistance, dysglycemia, and dyslipidemia (Eckel et al., 2010). It is associated with higher risks of cardiometabolic diseases, including coronary artery diseases and type 2 diabetes mellitus, and all-cause mortality (Gami et al., 2007). An increased prevalence of metabolic syndrome was confirmed in patients with psoriasis compared with general population in a meta-analysis of 12 observational studies (pooled OR: 2.26, 95% CI: 1.70–3.01) (Armstrong et al., 2013). A subsequent meta-analysis reached a similar conclusion after adjusting for confounders (pooled OR: 1.42, 95% CI: 1.28–1.65) (Rodríguez-Zúñiga and García-Perdomo, 2017), and showed that the risk of metabolic syndrome was closely related to psoriasis severity (Parodi et al., 2014). Dyslipidemia and hypertension are considered essential components of metabolic syndrome and had a higher prevalence among patients with psoriasis than in control groups in previous research (Kim et al., 2019; Snekvik et al., 2019). The potential risk of triggering psoriasis among patients with metabolic syndrome was found to increase in a prospective study; the association between individual components of metabolic syndrome and the incidence of psoriasis was also explored in this study (Kim et al., 2019). Some individual components such as a low level of high-density lipoprotein (HDL) cholesterol, a high level of triglycerides, and abdominal obesity promote psoriasis development (Snekvik et al., 2019). However, the conclusion about the impact of elevated blood pressure and fasting plasma glucose levels on the incidence of psoriasis remains controversial (Wu et al., 2014; Kim et al., 2017; Kim et al., 2019). Hence, more research is needed to determine the association between these factors.

### Effect of Tumor Necrosis Factor-*α* Inhibitors on Cardiometabolic Outcomes in Psoriasis

#### Effect on Cardiovascular Diseases

Psoriasis accompanied by the occurrence of high-risk cardiovascular events is associated with the spread of inflammation through blood vessels by the interactions of cytokines. Various studies have demonstrated that the risk of cardiovascular comorbidities in psoriasis patients reduced after treatment with TNF-*α* inhibitors. TNF-*α* has been identified as a pivotal cytokine in the pathogenesis of both atherosclerosis and autoimmune diseases such as rheumatoid arthritis (RA) and psoriasis (McKellar et al., 2009). Treatment with TNF-*α* inhibitors in psoriasis patients could reverse early atherosclerosis at the initial stage presented as significantly reduced arterial intima-media thickness (IMT) values without irreversible atherosclerotic plaque (which indicates the development of subclinical atherosclerosis) and decreased signal intensity on β-2-(18F)-fluoro-2-deoxy-D-glucose-Positron emission tomography/computed tomography (FDG-PET/CT), implying less vascular inflammation (Jókai et al., 2013; Dey et al., 2017). However, a randomized controlled trial (RCT) showed that the target-to-background ratio (TBR) of carotid vessel walls, an indicator of vascular inflammation, had a modest increase after adalimumab treatment for 52 weeks (Bissonnette et al., 2017). Anti-TNF-*α* therapy also has a favorable effect on the improvement of arterial stiffness, measured by the gold standard aortic pulse wave velocity (aPWV), in patients with psoriatic arthritis (Angel et al., 2010). Despite that a recent systematic review showed no significant effect of TNF-*α* inhibitors on the subclinical indicators of atherosclerosis in inflammatory diseases including psoriasis, a positive effect of TNF-*α* biologics on the clinical outcomes of CVDs *via* alternate pathways, such as primary disease remission or reduced prothrombotic tendency, cannot be ruled out (Knowles et al., 2020).

The cardioprotective effect of TNF-*α* inhibitors that reduce the risk of MI compared with topical agents has been demonstrated in a retrospective cohort study (Wu and Poon, 2013a; Wu and Poon, 2013b). Coronary microvascular dysfunction as a result of systematic inflammation in psoriasis patients was ameliorated manifested as an increase in the coronary flow reserve (CFR) from 2.2 ± 0.7 to 3.02 ± 0.8 (*p* < 0.0001) after anti-TNF-*α* therapy (Piaserico et al., 2016). Psoriasis patients receiving biologic agents including TNF-*α* inhibitors showed almost no difference in the progression of asymptomatic coronary artery diseases (CAD) in a follow up, while CT imaging data suggested a significant increase in the procession of CAD in the control group (Herédi et al., 2016). Another study confirmed a decreased burden of non-calcified coronary plaques after anti-TNF-*α* therapy compared with the patients who did not receive biologic treatment (*p* < 0.01) (Elnabawi et al., 2019a). Clinical or subclinical cardiac dysfunction such as left ventricular diastolic dysfunction and right ventricular systolic dysfunction is slightly more prevalent in patients with psoriasis, which has been uncovered to be ameliorated upon TNF-*α* inhibitor therapy (Ahlehoff et al., 2016; Herédi et al., 2016).

Whether the risk of heart failure reduces or increases in psoriasis patients treated with TNF-*α* inhibitors has been discussed for a long time. It has been reported that TNF-*α* has detrimental effects on chronic heart failure (CHF). However, there is no conclusive evidence to prove the specific therapeutic effect of TNF-*α* inhibitors on CHF in psoriasis patients (Hori and Yamaguchi, 2013). Multiple clinical trials have demonstrated that etanercept does not affect hospitalization or mortality due to CHF (Mann et al., 2004; Campanati et al., 2020). A high dose of infliximab exacerbates the CHF condition in psoriasis patients with CHF (Chung et al., 2003). The dose-dependent association between the deterioration of CHF and the application of TNF-*α* inhibitors has prompted the cautious usage of this agent in patients with CHF (Campanati et al., 2020). The New York Heart Association recommends that TNF-*α* inhibitors are contraindicated in patients with class 3 or 4 CHF as well as those with class 1 or 2 CHF whose ejection fraction is lower than 50% (Menter et al., 2008).

Psoriasis patients with cumulative exposure to TNF-*α* inhibitors for 6 months had more than 11.2% reduction of cardiovascular event risk compared with those who received phototherapy [hazard ratio (HR): 0.89, 95% confidence interval (CI): 0.79–0.99, *p* = 0.048] in a large-scale observational cohort study (Wu et al., 2018). A similar conclusion was reached in another study that the risk of cardiovascular events in patients receiving TNF-*α* inhibitors was lower than those receiving methotrexate (Wu J. J. et al., 2017). A meta-analysis of five studies including 49,795 patients with plaque psoriasis or psoriatic arthritis also verified the efficacy of anti–TNF-*α* therapy in decreasing the incidence of cardiovascular events (Yang et al., 2016). Another meta-analysis of 38 RCTs involving 18,024 patients treated with biologic therapy including TNF-*α* inhibitors reported 10 cases of MACEs in nine RCTs. However, a pooled analysis showed no significant statistical difference in the incidence of MACEs in patients treated with biologic therapy compared with those treated with conventional therapy or placebo (OR: 1.45, 95% CI: 0.34–6.24) (Rungapiromnan et al., 2017). Therefore, more RCTs are needed to provide the basis for the selection and rational usage of biologic agents in order to minimize the cardiovascular risk in patients with psoriasis (Gelfand, 2018).

#### Effects on Cardiovascular Biomarkers

The risk of CVDs, including coronary heart diseases and peripheral arterial diseases, can be predicted and characterized by cardiovascular biomarkers such as C-reactive protein (CRP) and vascular endothelial growth factor, which are related to systemic inflammation and endothelial dysfunction (Heeschen et al., 2003; Melander et al., 2009). The serum levels of these factors decreased after TNF-*α* inhibitor therapy for over 24 weeks in a prospective study (Boehncke et al., 2011). Another study investigated six additional cardiovascular risk markers, including vascular cell adhesion molecule-1 (VCAM-1), intercellular adhesion molecule-1, E-selectin, matrix metalloproteinase-9, myeloperoxidase, and total plasminogen activator inhibitor-1. They are linked to BMI and waist-hip ratio, and participate in the onset and development of cardiometabolic diseases, especially metabolic syndrome, in psoriasis patients (Sigurdardottir et al., 2014).

#### Effects on Metabolic Disorders

An association between weight gain and TNF-*α* inhibitor has been reported. And, different types of TNF-*α* inhibitors were associated with distinct characteristics of weight gain among the patients (Saraceno et al., 2008). The efficacy and response of TNF-*α* inhibitors with fixed-dose medications such as etanercept are impaired in obese individuals (Clark and Lebwohl, 2008). Furthermore, the results from a prospective study suggested that successful weight loss (≥5% from baseline values) by concomitant dietary intervention in obese patients resulted in a higher rate of disease improvement defined as minimal disease activity in patients with psoriatic arthritis (Di Minno et al., 2014). Therefore, in order to treat psoriasis and associated obesity, appropriate diet, physical exercise as well as weight loss are necessary to improve the therapeutic effect of biologic agents (Dalamaga and Papadavid, 2019). The clinical response to infliximab or ustekinumab is not affected by body weight in the treatment of psoriasis patients, because they are dosed in a weight-based manner (Clark and Lebwohl, 2008; Dalamaga and Papadavid, 2019).

A retrospective cohort study among RA and psoriasis patients revealed a decreased risk of diabetes mellitus among patients treated with TNF-*α* inhibitors compared with those treated with other nonbiologic agents (Solomon et al., 2011). Studies concerning the effects of etanercept on insulin sensitivity, which is a pivotal factor during the onset of metabolic syndrome and diabetes mellitus, show that etanercept has a positive effect on improving fasting glucose levels by attenuating insulin resistance (Marra et al., 2007; Stanley et al., 2011). Contradictory results have been reported in a RCT that the application of etanercept failed to change insulin secretion and sensitivity in psoriatic patients (Martínez-Abundis et al., 2007). Insulin sensitivity has been improved by another TNF-*α* inhibitor adalimumab in psoriasis patients without diabetes (Pina et al., 2015).

There is evidence that TNF-*α* inhibitors may have favorable effects on certain conditions of metabolic syndrome in the management of psoriasis. However, further exploration is needed to more precisely determine the influence (Channual et al., 2009). The impact of anti–TNF-*α* treatment on the lipid profile of psoriasis patients has not been concluded so far. Recently, a prospective cohort study reported that TNF-*α* inhibitors are beneficial for regulating the metabolic state by decreasing the levels of total cholesterol and low-density lipoprotein cholesterol (Botelho et al., 2020), but no significant difference occurred after adalimumab treatment in another study (Bissonnette et al., 2013).

### Effect of IL-12/23 Inhibitors on Cardiometabolic Outcomes in Psoriasis

Biologics targeting IL-23 include two types of monoclonal antibodies, namely anti–IL-12/23p40, including ustekinumab, briakinumab, and anti–IL-23p19, including guselkumab, tildrakizumab, and risankizumab. Multiple RCTs and pooled analyses have proven the safety of IL12/23p40 inhibitors; however, one of the anti–IL-12/23 compounds, briakinumab, was withdrawn from the market due to an increased cardiovascular risk since it caused frequent MACEs in the early phase (Gordon et al., 2012; Langley et al., 2013).

#### Clinical Evidence for the IL-12/23p40 Inhibitor Ustekinumab

The safety profile of another IL-12/23 inhibitor ustekinumab aroused concern after the withdrawal of briakinumab due to the high incidence of MACEs, and further studies aimed to evaluate the cardiovascular risk of this class of compounds. During a 3-years follow-up study, the combined MACE rate per 100 patient-years was 0.44 (95% CI: 0.27–0.67) in a pooled analysis of phase II/III clinical studies of ustekinumab on moderate-to-severe psoriasis, and the comparison of the standardized incidence ratios of psoriasis patients treated with ustekinumab with general population suggests that the effect of ustekinumab on MACEs is neither detrimental nor beneficial (Reich et al., 2011). A meta-analysis of 22 RCTs involving 10,183 psoriasis patients also showed no significant difference in the MACE rate between patients receiving anti–IL-12/IL-23 and placebo, with a Mantel-Haenszel risk difference of 0.012 events/person-year (95% CI: −0.001 to 0.026; *p* = 0.12) (Ryan et al., 2011), but a higher risk of MACEs in patients receiving IL-12/23 antibodies in comparison with placebo was found after the evaluation of the same trials using another statistical technique named the Peto OR method (Tzellos et al., 2013). The increased rate of MACEs in patients treated with anti–IL-23 therapy was confirmed in a meta-analysis of randomized clinical trials that mainly covered individuals with high cardiovascular risk (Ait-Oufella et al., 2019). A recent case-time-control study also identified a significant association between the initiation of treatment with ustekinumab and the early occurrence of severe cardiovascular events (OR: 4.17; 95% CI: 1.19–14.59) (Choi et al., 2020). Data from the PHOENIX 1 study of long-term ustekinumab treatment in patients with an extended duration of exposure showed a favorable safety profile and stable clinical response (Kimball et al., 2013). The influence of IL-12/23 inhibitors on cardiovascular safety was also compared with those of other biologics approved for the treatment of psoriasis in some trials, which reported a comparable risk of MACEs among ustekinumab, TNF-*α* inhibitors, and IL-17 inhibitors (Kimball et al., 2013; Ikonomidis et al., 2017; Gelfand, 2018). The risk of atrial fibrillation and MACEs did not substantially differ between treatment initiated with ustekinumab and TNF-*α* inhibitors in a cohort study (Lee et al., 2019a). However, in another analysis, ustekinumab showed a greater improvement in vascular, coronary and myocardial function, which was reflected in improved global longitudinal strain, left ventricular twisting, percent difference between peak twisting and untwisting at mitral valve opening (%untwMVO), and CFR as well as reduced circulating N-terminal pro-B-type natriuretic peptide (NT-proBNP) levels (Ikonomidis et al., 2017). To investigate the effect of biological therapy on the characteristics of coronary plaque phenotypes that partly determine the risk of MI, 290 participants treated with biologics were included in a 1-year follow-up study performed with serial coronary computed tomography angiography and the collection of clinical and laboratory data, and an improvement in the high-sensitivity-c-reactive protein (hs-CRP) level and a reduction in the non-calcified plaque burden were observed in the anti–IL-12/23 treatment group, which was inferior to the effects of anti-TNF-*α* and anti–IL-17 in the prospective, observational study (Hjuler et al., 2016). The incidence of a lipid-rich necrotic core, which is a high-risk coronary plaque feature, was also found to decrease after 1 year of biological therapy, including anti–TNF-*α*, anti–IL-12/23, and anti–IL-17 therapy, compared with the nonbiologic therapy group (Poizeau et al., 2020). Similarly, anti–IL-12/23 and anti–IL-17 therapy was associated with a significant reduction in coronary inflammation measured by the perivascular fat attenuation index in a prospective cohort study (Hjuler et al., 2016).

Although anti–TNF-*α* therapy appears to increase the weight and BMI of patients, this effect does not appear in patients treated with anti–IL-12/23 therapy with weight-adjusted doses, suggesting that ustekinumab could be considered to treat overweight and obese patients with psoriasis (Wu et al., 2020). However, the significance of weight loss during the biological treatment of obese patients cannot be ignored because multiple studies have proven that a reduction in weight improves the clinical response to ustekinumab (Zhu et al., 2009; Gisondi et al., 2016). No significant change in the mean lipid levels after the biological therapy, including ustekinumab or placebo, for 1 year at follow-up was observed in another study (Hjuler et al., 2016). Similar results were also observed for BMI, lipid and glucose levels, which remained unchanged at the 1-year follow-up assessment of psoriasis patients treated with biologics (Elnabawi et al., 2019b).

#### Ongoing Research About IL-23p19 Inhibitors

Agents targeting the p19 subunit of the IL-23 cytokine pathway have been approved for treatment only for 3 years, so the data for the incidence of MACEs in the clinical trials for the safety profile of IL-23p19 inhibitors is not sufficient to determine its influence on the cardiovascular risk of psoriasis patients (Blauvelt et al., 2017; Papp et al., 2017). Three completed phase 3 trials of guselkumab in the treatment of psoriasis, including VOYAGE 1 (NCT02207231), VOYAGE 2 (NCT02207244), and NAVIGATE (NCT02203032) reported low rates (<1.5%) of MACE occurrence over the study period in all groups (Blauvelt et al., 2017; Reich et al., 2017; Langley et al., 2018). A pooled analysis of three RCTs on tildrakizumab, including a phase 2b study (NCT01225731) and reSURFACE 1 (NCT01722331) and 2 (NCT01729754), also reported low rates of MACEs (Blauvelt et al., 2018). A low incidence of MACE was also reported in two phase 3 trials of risankizumab vs ustekinumab: UltIMMa‐1 (NCT02684370) and UltIMMa‐2 (NCT02684357) (Gordon et al., 2018). A total of 109 studies on the efficacy and safety of the therapy for psoriasis involving biologic agents were included in a network meta‐analysis suggesting that no significant difference exists in the risk of MACE occurrence among guselkumab, tildrakizumab, ustekinumab, certolizumab, infliximab, adalimumab, and etanercept, versus placebo (Sbidian et al., 2017). More data are needed to confirm the effect of the anti–IL-23p19 agents on cardiovascular and metabolic risk.

### Relationship Between IL-17 Inhibitors and Cardiovascular Risk in Psoriasis

#### IL-17 and Atherosclerosis

The existing data evaluating the effects of IL-17 inhibitors as newly-approved clinical biologic agents targeting IL-17 signaling on the risk of CVDs in psoriasis patients are insufficient. However, experimental data have shown that overexpression of IL-17A in keratinocytes of the murine model [K14-IL-17A (ind/+)] induced systemic vascular inflammation, arterial hypertension, and endothelial dysfunction, all of which can lead to an increased risk of CVDs (Karbach et al., 2014). IL-17A, which is involved in the development of psoriasis, is also implicated in the pathogenesis of CVDs, which suggests that IL-17A-mediated inflammation may become a potential overlapping pathological mechanism between psoriasis and its cardiovascular comorbidities (Lockshin et al., 2018). Research on atherosclerosis yielded conflicting results, suggesting that the IL-17 mainly produced by Th17 cells may promote or prevent atherosclerotic plaque development and stability, which are determined by specific inflammatory condition (Taleb et al., 2015). Atherosclerotic plaque stability is enhanced by the cytokine profile of increased IL-17 levels and decreased level of interferon-*γ* (IFN-*γ*) in the local microenvironment, whereas the increased production of IL-17 and IFN-*γ* has a synergistic pro-inflammatory effect in promoting disease progression (Taleb et al., 2015). Other studies also suggested that IL-17 maintained the stability of atherosclerotic plaques by promoting the generation of collagen and smooth muscle cells and downregulating the level of VCAM-1 (Taleb et al., 2009; Gisterå et al., 2013). However, the findings regarding the percentage of circulating Th17 cells and related cytokine IL-17A in patients with acute coronary syndromes are discordant that some reported increasing levels and others showed no significant difference in comparison with patients without coronary artery diseases (Cheng et al., 2008; Eid et al., 2009). The association between IL-17-mediated inflammation and atherosclerotic plaque conformation as well as the instability has yielded evidence for the hypothesis that psoriasis patients have a high risk of MI (Chen et al., 2010). Given the association between IL-17 and atherosclerosis in psoriasis, the effects of IL-17 inhibitors on cardiovascular comorbidities in the treatment of psoriasis should be evaluated carefully.

#### IL-17 Inhibitors and Cardiovascular Events

In phase III clinical trial programs of IL-17 inhibitors, a small section of patients experienced a few cases of MACEs (Langley et al., 2014; Griffiths et al., 2015; Lebwohl et al., 2015). Low levels of IL-17 in patients has been shown to be associated with a higher risk of recurrent MI and death in the related research (Simon et al., 2013). In a meta-analysis including 5,951 patients from 9 RCTs that compared the efficacy and safety of IL-17 inhibitors with placebo, six cases of MACEs were reported in 2,143 patients treated with IL-17 inhibitors whereas 700 patients treated with placebo reported only one MACE across four studies; however, no significant difference was observed between IL-17 inhibitors and placebo (Wu D. et al., 2017). Another meta-analysis of the effect of biologic therapy on the risk of MACEs showed 2 MACEs in 514 patients receiving IL-17 inhibitors among 8 MACEs in patients treated with various biologics (*n* = 12,596) and 2 MACEs in the placebo group (*n* = 5,092), and the pooled analysis of biologics and placebo, as well as various separate agents including TNF-*α* inhibitors, anti–IL-17A agents, or anti–IL-12/23 agents, did not show a significant difference in the risk of MACEs (Rungapiromnan et al., 2017). In a prospective, observational study of 215 psoriasis patients receiving biologic therapy recruited over a 1-year follow-up, an improvement in hs-CRP and HDL cholesterol level was observed in the anti–IL17-treated groups, and the most significant reduction (up to 12%) in coronary plaque burden appeared in the anti–IL-17 therapy group among all biologic-treated groups (Elnabawi et al., 2019a). Exposure-adjusted incidence rates of cardiovascular events were comparable between the secukinumab (300 mg/150 mg) group and etanercept group in the pooled analysis of 10 clinical trials over 52 weeks in 3,993 psoriasis patients (van de Kerkhof et al., 2016). In phase III clinical trials of brodalumab AMAGINE-1, five MACEs occurred, including two in patients treated with placebo and three in patients receiving constant 210 mg of brodalumab (Papp et al., 2016). Moreover, in the AMAGINE-2 and AMAGINE-3 studies, deaths occurred due to cardiovascular problems such as stroke and cardiac arrest (Lebwohl et al., 2015). A study evaluating the long-term safety of ixekizumab from 13 clinical trials reported 84 cases of MACEs among 5,697 psoriasis patients, with no significant increase in the cardiovascular risk or the incidence (Armstrong et al., 2020). Overall, data obtained primarily from pivotal clinical trials of short-term studies on anti–IL-17 therapy in patients with moderate-to-severe psoriasis plaque did not demonstrate an increased risk of CVDs. However, considering the limitations of these data, more long-term studies are necessary to determine the cardiovascular risk.

Prospective CARIMA trials (NCT02559622) that incorporated patients with moderate‐to‐severe psoriasis with or without severe CVD aimed to evaluate the influence of secukinumab on endothelial dysfunction over 52 weeks, and indicated a protective effect of secukinumab on CVD by improving the endothelial function and flow-mediated dilation in psoriasis patients (von Stebut et al., 2019). Recently prospective studies on the impact of IL-17 inhibitors on cardiovascular risk are ongoing such as Vascular Inflammation in Psoriasis (VIP) trials of secukinumab (VIP‐S; NCT02690701), which is evaluating the cardiovascular risk in psoriasis patients from many aspects, including aortic inflammation and cardiometabolic biomarkers.

#### IL-17 Inhibitors and Obesity

Inflammation propagation in obese adipose tissues is facilitated by IL-17A without impairing the adipogenesis and insulin response induced by the inflammatory environment (Pestel et al., 2020). In real-life cohorts including a majority of psoriasis patients with cardiometabolic comorbidities, real-world data of the baseline characteristics and clinical response to secukinumab suggested that patients with high BMI and obesity appeared to show a lower persistence of the curative effect and an adverse effect of PASI ≤3 response at week 78 (Rompoti et al., 2020). In contrast to the weight-enhancing effects of TNF-*α* inhibitor treatment, anti–IL-17 therapy as well as anti–IL-12/23 therapy appears to show no increase in body weight and BMI (Wu et al., 2020). Risk-benefit analyses of four RCTs that enrolled 2,403 patients with plaque psoriasis showed that patients with body weight less than 90 kg treated with 150 mg secukinumab and patients with body weight of 90 kg or more treated with 300 mg secukinumab had comparable efficacy and safety, suggesting that weight is a pivotal factor to be considered in dosing regimen recommendation (Lee et al., 2019b). However, another IL-17 inhibitor brodalumab showed no significant difference in efficacy and safety between nonobese and obese patients in post-hoc analysis of the AMAGINE-2 and AMAGINE-3 trials (Hsu et al., 2020). The impact of the IL‐17A inhibitor secukinumab on adipose tissue and cutaneous inflammation in patients with moderate‐to‐severe psoriasis is being explored prospectively in the ongoing ObePso‐S trial (NCT03055494) (Korman, 2020).

The findings from studies exploring the effect of biologic therapy on cardiometabolic syndrome in the treatment of psoriasis are summarized in Table 1.

**TABLE 1:** Effect of biologics on cardiometabolic comorbidities.

Target therapy	Biologics	Effect of biologics on			
		Cardiovascular diseases	Obesity	Metabolic syndrome	Diabetes mellitus
TNF-*α* inhibitors	Adalimumab infliximab certolizumab-pegol etanercept	Various studies have demonstrated a reduced cardiovascular risk Wu and Poon (2013a), Wu and Poon (2013b), decreased level of cardiovascular bio-markers Boehncke et al. (2011), Sigurdardottir et al. (2014), and improved arterial stiffness after treatment Angel et al. (2010). Controversy exists in the effect of vascular inflammation Bissonnette et al. (2013), Bissonnette et al. (2017), Dey et al. (2017).	Observational data indicated an increase in weight gain after treatment Saraceno et al. (2008). A prospective study indicated a better efficacy and response of biologics after weight loss Di Minno et al. (2014). Infliximab (dosed on weight) is not affected by weight Clark and Lebwohl (2008), Dalamaga and Papadavid (2019).	A prospective cohort study reported TNF-*α* inhibitors are beneficial to regulate metabolic state by decreasing levels of total cholesterol and low-density lipoprotein cholesterol Botelho et al. (2020), but a more precise impact needs further exploration Channual et al. (2009).	Contradictory results have been reported that the effect of etanercept on insulin sensitivity Marra et al. (2007), Martínez-Abundis et al. (2007), Stanley et al. (2011). Adalimumab improved insulin sensitivity in non-diabetic patients affected by psoriasis in a prospective study Pina et al. (2015).
IL-12/23p40 inhibitors	Ustekinumab briakinumab	Briakinumab was withdrawn from the market due to the increased cardiovascular risk Gordon et al. (2012), Langley et al. (2013). The effect of ustekinumab on MACEs demonstrated in several studies is neither detrimental nor beneficial Reich et al. (2011), Ryan et al. (2011). Another analysis reported a greater improvement of vascular, coronary, and myocardial function after ustekinumab treatment Ikonomidis et al. (2017).	Anti-IL-12/23 therapy appears no increase in body weight and BMI Wu et al. (2020).	No significant change of mean lipid levels after ustekinumab treatment for 1 year at follow-up was observed in a study Hjuler et al. (2016). And similar results were also found in another study that body mass index, lipids, or glucose remained unchanged at 1-year follow-up of psoriasis treated with biologics Elnabawi et al. (2019b).	
IL-17 inhibitors	Ecukinumab ixekizumab brodalumab	IL-17 has the double effect of promoting or preventing atherosclerotic plaques Taleb et al. (2015). IL-17 therapy didn’t demonstrate an increased risk of cardiovascular diseases in a few short-term studies Wu et al. (2017b), Rungapiromnan et al. (2017), Armstrong et al. (2020). More long-term prospective studies are ongoing.	IL-17 inhibitors appear no increase in body weight and BMI Wu et al. (2020). Weight is a pivotal factor to influence the efficacy and response of anti-IL17 therapy Lee et al. (2019b), Rompoti et al. (2020).	—	—
IL-23p19 inhibitors	Guselkumab tildrakizumab risankizumab	Current clinical trials are not sufficient to draw conclusion about its impact on cardiometabolic risk of psoriasis patients, only can reflect the cardiovascular safety in a short term.			

### Overlapping Mechanisms Between Cardiometabolic Diseases and Psoriasis

**FIGURE 1:**
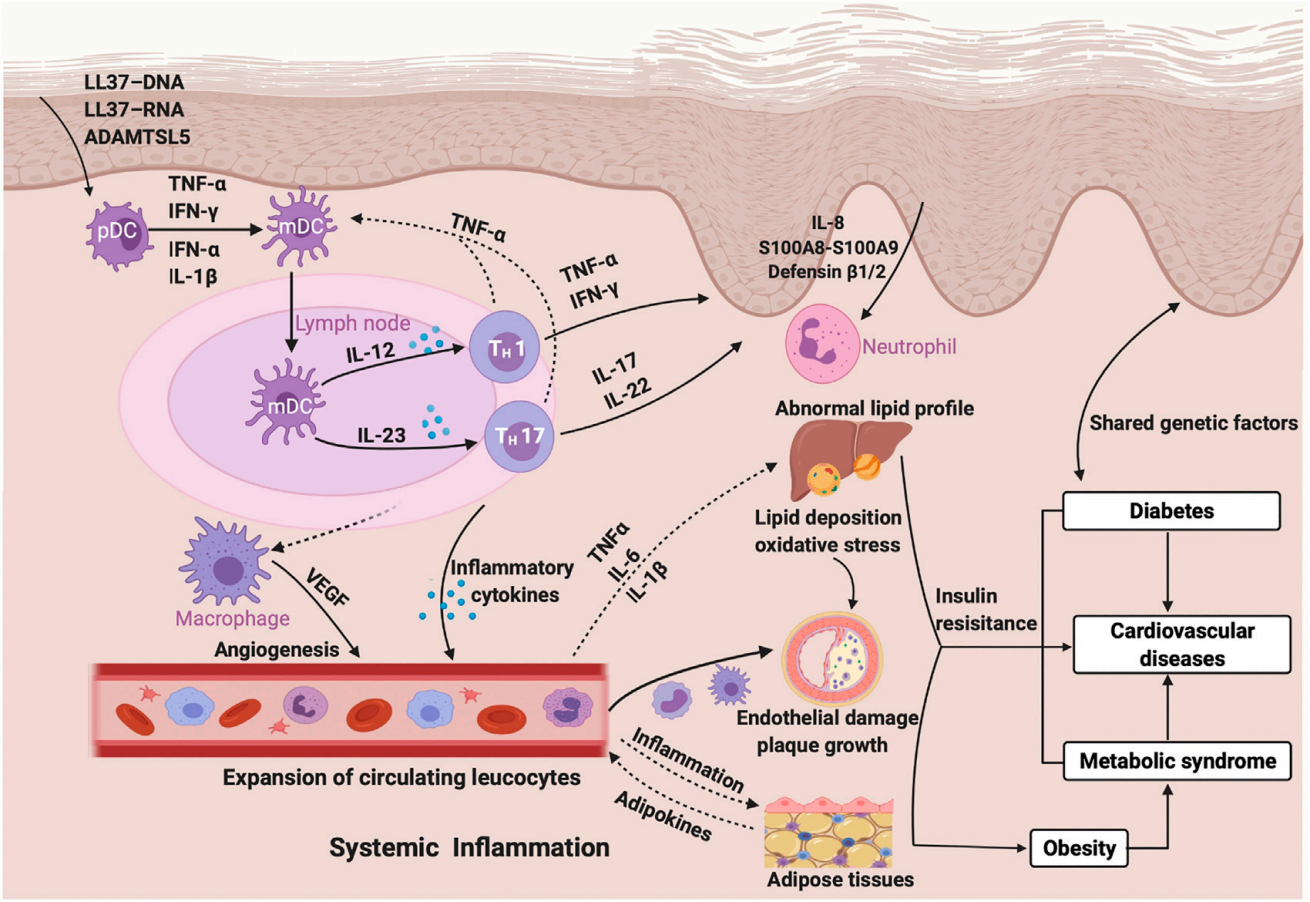
Psoriasis, cardiometabolic comorbidities, and their common inflammatory pathway.

Laboratory research has explored mechanistic links between psoriasis and CVDs related to the inflammatory cascades integrated by activated immune cells and upregulation of proinflammatory cytokines and mediators that influence both psoriasis and atherosclerosis (Flammer and Ruschitzka, 2012). Activation of the innate immune system is considered to be indispensable to initiate the inflammatory cascade in psoriasis, and components of the innate immune system, including neutrophils, dendritic cells, macrophages, and pro-inflammatory cytokines such as TNF-*α* and IL-18 have been shown to exist in psoriatic adipose tissue. Deranged lipid distribution and impaired adipose function have been confirmed to accelerate the build-up of atherosclerotic plaques in cardiovascular diseases (Sajja et al., 2018). The critical T-cell differentiation into Th1 and Th17 cells, which is stimulated by IL-12 and IL-23 and leads to the release of cytokines such as TNF-*α*, IFN-*γ*, IL-17, and IL-22 in the process of adaptive immunity, overlaps between psoriasis and cardiometabolic diseases leading to augmented keratinocyte proliferation and angiogenesis (Lockshin et al., 2018). Another potential mechanism of insulin resistance can be induced by the chronic inflammatory state in psoriatic diseases, and both of them may contribute to the early stage of the formation of atherosclerotic plaques leading to cardiometabolic diseases by causing endothelial dysfunction and increased intima-media thickness in patients with psoriasis (Siegel et al., 2013). Adipokines, a group of proteins secreted by adipocytes, including leptin, visfatin, and resistin, promote the inflammatory condition in psoriasis patients by the correlation with immune cells and pro-inflammatory factors, which in turn results in the appearance of an abnormal serum adipokine profile in patients with psoriasis (Toussirot et al., 2014). Genetic and environmental factors play a role in the comorbidity of psoriasis and metabolic disorders (Lønnberg et al., 2016). For example, the pleiotropic susceptibility loci CDKAL1 contributes to the occurrence of psoriasis as well as diabetes mellitus (Wolf et al., 2008), which may upregulate the inflammatory cytokines in psoriasis promoting insulin resistance, an independent risk factor of T2DM (Gelfand, 2016). One study indicated that psoriatic patients with normal glucose tolerance show insulin resistance or impaired insulin sensitivity, which may further develop into diabetes (Gyldenløve et al., 2015). The inflammatory condition affecting the systemic circulation in psoriasis is regarded as a promoter of components of metabolic syndrome such as insulin resistance, vascular dysfunction, dyslipidemia, and the systemic inflammation also becomes an inducer of endothelial dysfunction, oxidative stress, and increased angiogenesis (Gisondi et al., 2018). Therefore, systemic inflammation may be the foremost shared pathophysiology pathways between psoriasis and cardiometabolic diseases, which awaits future exploration (Figure 1).

### Suggestions for the Management of Cardiometabolic Diseases in Psoriasis

Cardiovascular risks in psoriatic patients should be addressed with vigilance, and screening for the traditional cardiovascular risk factors and inflammatory markers such as CRP should be performed to predict the incidence of cardiovascular events (Kimball et al., 2008). Appropriate treatment options should be implemented considering the systemic condition of patients to reduce the risk and severity of cardiovascular disorders (Kristensen et al., 2015). Early determination of the cardiovascular risk in patients with psoriasis is recommended to facilitate effective management and prevent the occurrence of cardiovascular events in psoriatic patients, and precautions against cardiovascular events may also reduce the severity of psoriasis (Hu and Lan, 2017). The significance of weight control and monitoring should be emphasized because weight loss can improve the severity of psoriasis in obese patients and decrease the risk of cardiovascular events (Debbaneh et al., 2014). In the majority of studies, weight or BMI was a critical factor that negatively affected the therapeutic response to biologics and disease-modifying anti-rheumatic drugs; therefore, more attention should be paid to weight or BMI control during the treatment (Batalla et al., 2015). Screening the risk of diabetes mellitus in patients with psoriasis and implementation of preventive measures against the occurrence of diabetes mellitus should also receive importance, especially in patients with high affected BSA, since data shows that every 10% increase in BSA affected by psoriasis is accompanied by a 20% increase in diabetes risk (Wan et al., 2018). During the treatment of psoriasis, attention should be paid to the relationship between concomitant medications and the hazard of diabetes mellitus to select appropriate drugs for treatment, and blood glucose monitoring of patients should be strengthened (Lee et al., 2014). Given the mutual interaction of psoriasis and metabolic syndrome, preventive management of individual components of metabolic syndrome can also prevent the inflammation infiltrating into the skin to cause psoriasis. On the other hand, the significance of monitoring blood pressure, fasting plasma glucose level, HDL cholesterol, triglyceride level, and waist circumference to determine the development of metabolic syndrome after the diagnosis of psoriasis cannot be ignored. It is worth mentioning that there are certain other comorbidities and concomitant conditions, such as pediatric age group, pregnancy, and concomitant chronic infections, in addition to cardiometabolic comorbidities discussed in this review. All the comorbidities and concomitant conditions should also be considered when selecting optimal treatment for psoriasis patients to guarantee its safety and efficacy (Kaushik and Lebwohl, 2019a; Kaushik and Lebwohl, 2019b).
